# The Minimal Important Difference in Physical Activity in Patients with COPD

**DOI:** 10.1371/journal.pone.0154587

**Published:** 2016-04-28

**Authors:** Heleen Demeyer, Chris Burtin, Miek Hornikx, Carlos Augusto Camillo, Hans Van Remoortel, Daniel Langer, Wim Janssens, Thierry Troosters

**Affiliations:** 1 KU Leuven-University of Leuven, Department of Rehabilitation Sciences, B-3000 Leuven, Belgium; 2 University Hospitals Leuven, Department of Respiratory Diseases, B-3000 Leuven, Belgium; 3 Center for research in environmental epidemiology (CREAL), Barcelona, Spain; 4 Rehabilitation Research Centre, Biomedical Research Institute, Faculty of Medicine and Life Sciences, Hasselt University, Diepenbeek, Belgium; 5 University Hospitals Leuven, Department of Cardiovascular Sciences, B-3000 Leuven, Belgium; 6 Red Cross Flanders, Centre for Evidence-Based Practice, Mechelen, Belgium; Pondicherry Institute of Medical Sciences, INDIA

## Abstract

**Background:**

Changes in physical activity (PA) are difficult to interpret because no framework of minimal important difference (MID) exists. We aimed to determine the minimal important difference (MID) in physical activity (PA) in patients with Chronic Obstructive Pulmonary Disease and to clinically validate this MID by evaluating its impact on time to first COPD-related hospitalization.

**Methods:**

PA was objectively measured for one week in 74 patients before and after three months of rehabilitation (rehabilitation sample). In addition the intraclass correlation coefficient was measured in 30 patients (test-retest sample), by measuring PA for two consecutive weeks. Daily number of steps was chosen as outcome measurement. Different distribution and anchor based methods were chosen to calculate the MID. Time to first hospitalization due to an exacerbation was compared between patients exceeding the MID and those who did not.

**Results:**

Calculation of the MID resulted in 599 (Standard Error of Measurement), 1029 (empirical rule effect size), 1072 (Cohen's effect size) and 1131 (0.5SD) steps.day^-1^. An anchor based estimation could not be obtained because of the lack of a sufficiently related anchor. The time to the first hospital admission was significantly different between patients exceeding the MID and patients who did not, using the Standard Error of Measurement as cutoff.

**Conclusions:**

The MID after pulmonary rehabilitation lies between 600 and 1100 steps.day^-1^. The clinical importance of this change is supported by a reduced risk for hospital admission in those patients with more than 600 steps improvement.

## Introduction

Physical inactivity is an important predictor of worse outcome in Chronic Obstructive Pulmonary Disease (COPD), resulting in an increased risk of hospital admission and mortality [1]. Patients with COPD are less active compared to age matched controls and this inactivity worsens with increasing disease severity [2]. Independent of the disease severity, physical activity decreases over time and a sustained physical inactivity is associated with a progression of exercise intolerance and muscle depletion [3]. Therefore, assessing and increasing physical activity (PA) has gained importance in COPD management [2]. Objective measures of PA do not rely on information provided by the patient and can give a valid presentation of the physical activity level in this slow walking population [4,5]. This gives the ability to investigate the effect of interventions on the PA level. Pulmonary rehabilitation, the most effective non-pharmacological treatment, increases PA to a statistically significant extent [6], equivalent to five minutes more activity per day [7]. The interpretation of the increase seen after pulmonary rehabilitation remains difficult as the minimal important difference (MID) has not yet been established [8].

The Food and Drug administration (FDA) defines the MID as “a meaningful change or effect that might be considered important, beneficial or harmful, either by patients or informed proxies (including clinicians) and which would lead the patient/clinician to consider a change in management”[9]. The MID has become the standard approach for the interpretation of clinically relevant changes of an intervention and is well-established to guide changes in treatment decisions (by clinicians) and to calculate sample sizes (by investigators) [10].

The MID can be derived from distribution-based methods by observing changes in a database and interpreting results in terms of the relationship between the magnitude of the effect and measures of variability. A second technique are anchor-based approaches which associate outcomes with related concepts [9]. Because none of the approaches is perfect, the recommendation is to estimate the MID based on several anchor- and distribution-based methods and add relevant clinical or patient-based indicators and to triangulate to a single value or a small range of values for the MID [11].

Testing whether the MID estimation based on the triangulation process is important can be done by comparing the MID with acceptable patient-reported outcomes or clinical end-points. In patients with COPD, acute exacerbations are independent indicators of poor prognosis [12]. Reducing the frequency and severity of exacerbations is one of the priorities in the treatment of stable patients with COPD [13]. Patients with lower levels of PA are hospitalized more rapidly [14] and have more exacerbations [15,16] compared to more active patients. Moreover, based on self-report, an increase in physical activity resulted in a lower frequency of hospitalizations for an exacerbation [17]. It could, hence, be speculated that patients with an increase exceeding the MID should experience less severe exacerbations after pulmonary rehabilitation.

The aims of the present study were 1) to provide an estimate of the MID in physical activity (step count) in patients with COPD and 2) to validate the clinical importance of this MID by investigating the time to the first admission for an exacerbation in the 2 years following a rehabilitation program.

## Methods

### Study population and design

This retrospective study has been approved by UZ Leuven Medical Ethics Committee (S58489). No informed consent from the participants was required for the present analysis. All patient information was anonymized and de-identified prior to analysis. Eighty nine patients formed the ‘rehabilitation sample’ and included 57 patients from an earlier study [18] as well as 32 newly consecutive recruited patients from this rehabilitation program between November 2011 –February 2013, in whom PA measurement was available as part of the clinical routine assessment. An additional and independent ‘test-retest sample’ of 37 patients was recruited from the multicenter PROactive trials [4,19], using patients included in our center. This cohort was included to analyze test-retest reliability of two consecutive weeks of measurement, as this information was not available in literature.

Inclusion criteria and content of this outpatient multidisciplinary rehabilitation are described elsewhere [20,21].

### Clinical measurements

PA was measured by the Sensewear Pro Armband (BodyMedia, Pittsburgh, PA, USA) or the Actigraph GT3X (Actigraph LLC Pensacola, FL, USA), both accelerometers validated in patients with COPD [4,5]. Day-by-day differences in steps were comparable between both monitors (S1 File). In the rehabilitation sample PA was measured for one week before and one week immediately after three months of rehabilitation. Patients in the test-retest sample were measured for two consecutive weeks. Weekends and weekdays with less than eight hours of wearing time were excluded from the analysis in both cohorts [18]. Patients (in both cohorts) were excluded if they did not have at least four valid weekdays of measurement, in both weeks. The mean number of steps per week was chosen as PA outcome.

In both samples lung function (Jaeger Master Screen Body; CareFusion; Germany) was measured according to ERS/ATS standards [22]. The results were expressed as predicted normal values [23]. In the rehabilitation cohort two six-minute walking tests (functional exercise capacity) and the Chronic Respiratory Disease Questionnaire (CRDQ) (health-related quality of life) [24] were performed at baseline and after finishing the program. The dyspnea subscale (CRDQ_dyspnea_) and total score (CRDQ_total_) were retrieved for the present investigation.

### Statistical analysis

All statistical analyses were performed using the SAS statistical package (v9.3,SAS, institute,Cary,NC,USA). P<0.05 was considered as statistically significant.

### Calculation of the MID

The distribution-based techniques used are 1) standard error of measurement (SEM), 2) empirical rule effect size, 3) cohen’s effect size and 4) 0.5 times the standard deviation (SD) of the baseline measurement (Table 1). In the calculation of cohen’s effect size and the empirical rule effect size, the SD of the change score was used [25]. PA changes in the rehabilitation sample were adjusted for daylight time between the measurement before and after rehabilitation (PROC MIXED analysis including daylight as covariate) [18]. The intraclass correlation coefficient (ICC) was calculated based on the test-retest cohort (PROC MIXED).

**Table 1:** Distribution-based estimates of the minimal important difference (MID).

Method	MID calculation
**SEM**	**SD**_**baseline**_ *sqrt (1-ICC)
**Empirical rule effect size**	0.08 * 6 * SD_**∆**_
**Cohen’s effect size**	0.5*SD_**∆**_
**0.5 times SD**	0.5*SD_**baseline**_

SEM = standard error of measurement, SD = standard deviation (based on rehabilitation sample), sqrt = square root, ICC = intraclass correlation coefficient (based on test-retest sample), Δ = difference in steps (based on rehabilitation sample), baseline = number of steps at baseline (based on rehabilitation sample)

Anchor-based methods have two requirements, 1) the anchor must be interpretable and 2) there must be an appreciable association between the measurement of interest and the anchor [10] (correlation of ≥0.5 [25]). The six-minutes walking distance (6MWD) and CRDQ are associated with the level of PA in cross sectional analyses [1] and changes in CRDQ_dyspnea_ and 6MWD have been (although not strongly) related to changes in walking time after rehabilitation [21]. Since the MID for the 6MWD, CRDQ_total_ score and the CRDQ_dyspnea_ domain are well established [8], these were chosen as possible anchors. In the presence of a sufficient (pearson) correlation, a linear regression analysis provides the estimation, using a change of 30 meters in the 6MWD, 2.5 points in the CRDQ_dyspnea_ and 10 points for CRDQ_total_ score [8].

### Including a relevant clinical indicator

Time to first hospital admission for an exacerbation in the two years following the three months of rehabilitation was collected (data collection until 1^st^ of April 2014).

A priori reasons for censoring were a restart in a rehabilitation program, transplantation, cancer, a cerebrovascular accident, severe cardiovascular events (need for ICU stay) and loss of contact with the patient. No censoring was done for mild (without stay in the ICU) cardiovascular events.

Kaplan-Meier curves of time to first admission during follow up were built representing PA of patients exceeding the MID and those who didn’t, using each of the calculated MIDs. Differences were analyzed using a Cox proportional hazard regression analysis (proc phreg) with each calculated MID as class variable. Statistical differences and the hazard ratios (+95% confidence intervals) between patients exceeding the MID and patients who don’t were retrieved. In an additional analysis, the baseline PA (Steps_baseline_) and disease severity (FEV_1_%pred_baseline_) were included in the regression analysis to verify whether the difference was not solely explained by these important predictors of hospitalization risk.

## Results

### Patient characteristics

Fifteen patients in the rehabilitation sample were excluded because of an insufficient number of valid days. In the test-retest sample seven patients were excluded based on the PA criteria. There was no baseline difference between included and excluded patients, in both cohorts (S1 File). The rehabilitation (n = 74) and test-retest (n = 30) samples were comparable at baseline (Table 2).

**Table 2:** Baseline characteristics.

Parameter	Rehabilitation sample (n = 74)	Test-retest sample (n = 30)	p-value^$^
**Age (years)**	**66 ± 7**	**67 ± 7**	**0.36**
**BMI (kg.m**^**-2**^**)**	**26 ± 6**	**27 ± 6**	**0.49**
Gender (Male)*	**56 (76)**	**23 (77)**	**0.91**
**6MWD (m)**	**409 ± 120**	**457 ± 113**	**0.06**
**FEV**_**1**_ **(%**_**pred**_**)**	**48 ± 22**	**48 ± 12**	**0.93**
**Steps (n.day**^**-1**^**)**	**3839 ± 2262**	**4230 ± 2093**	**0.42**

Data expressed as mean ± SD

* = data expressed as n (%)

$ = between group differences analysed using an unpaired ttest or chi-square test(*)

### Calculation of the MID

Patients, respectively in the test-retest and rehabilitation sample, wore the monitor for a mean±SD of 4.9±0.4 weekdays with a wearing time of 962±233 minutes and 4.8±0.4 weekdays with a wearing time of 873±210 minutes.

The ICC between test and retest was 0.93 (daily steps week_1_ 4230±2093, week_2_ 4060±2148, p = 0.76). Patients in the rehabilitation cohort significantly increased their PA level with 805±2144 steps (p = 0.002), adjusted for a difference in daylight time (mean 0.7±274 minutes).

Table 3 summarizes the different MID estimations using distribution-based techniques. The MID ranges between 599 and 1131 steps. Unadjusted estimates only differed minimally from those including daylight time as covariate.

**Table 3:** Distribution-based methods to estimate the minimal important difference (MID) in physical activity.

Method	MID (steps.day^-1^)
**SEM**	**599**
**Empirical rule effect size**	**1029**
**Cohen’s effect size**	**1072**
**0.5 times SD**	**1131**

SEM = standard error of measurement, SD = standard deviation

**Fig 1:**
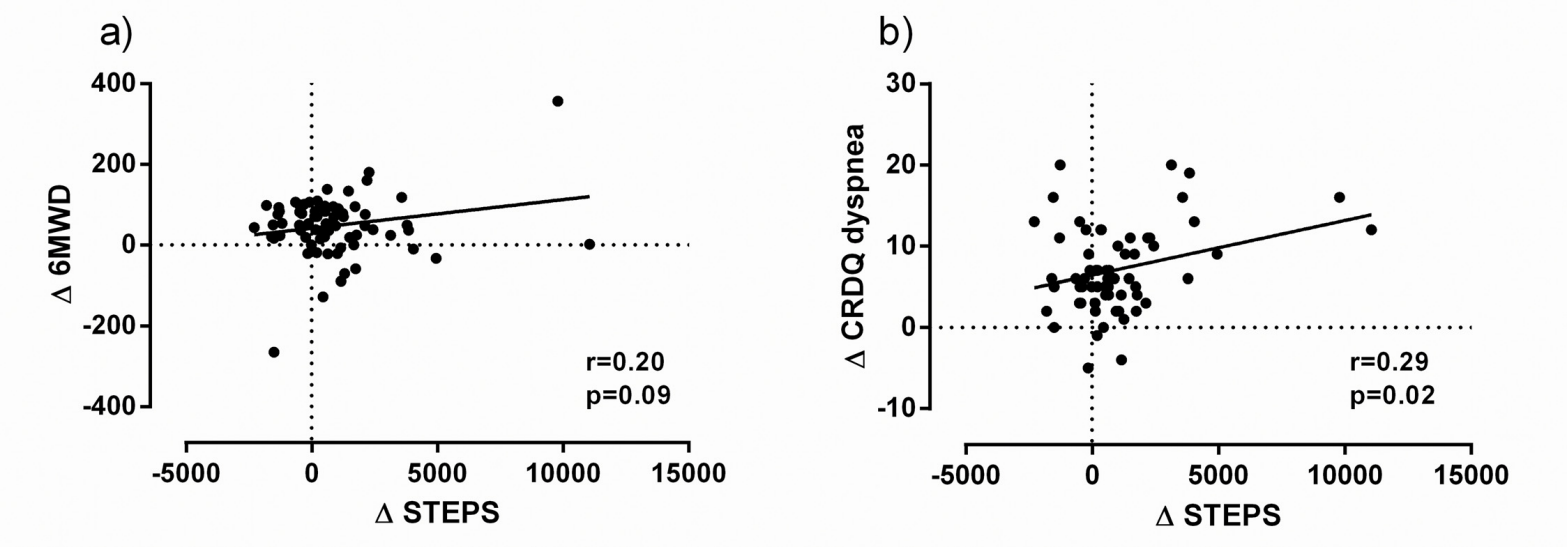
Correlation between change in daily step count and possible anchors. a) 6MWD and b) CRDQ_dyspnea_ in the rehabilitation sample (n = 74); 6MWD after 3 months was missing in 3 patients, CRDQ_dyspnea_ scores were missing in 10 patients.

Patients significantly improved their functional exercise capacity and CRDQ score (Table 4). Neither the change in 6MWD (r = 0.20, p = 0.09), nor the change in CRDQ_dyspnea_ score (r = 0.29, p = 0.02) or CRDQ_total_ score (r = 0.16, p = 0.27) were moderately correlated with the change in PA and could therefore not be used as reliable anchors (Fig 1). Excluding the outliers did not improve the presented correlations.

**Table 4:** Benefits after rehabilitation (rehabilitation sample, n = 74).

Parameter	Pre rehabilitation	Post rehabilitation	∆	p-value^$^
**Steps (n.day**^**-1**^**)**	**3839 ± 2262**	**4644 ± 3003**	**805 ± 2193**	**0.002**
**6MWD (m)**	**409 ± 120**	**459 ± 114**	**48 ± 76**	**<0.001**
**CRDQ**_**dyspnea**_	**15 ± 5**	**22 ± 6**	**7 ± 5**	**<0.001**
**CRDQ**_**total**_	**77 ± 14**	**97 ± 15**	**19 ± 13**	**<0.001**

Data expressed as mean ± SD; CRDQ_dyspnea_ baseline was missing in 5 patients, 6MWD at 3 months was missing in 3 patients, CRDQ_dyspnea_ at 3 months was missing in 10 patients; CRDQ_total_ score reported based on 50 patients; ∆steps data present unadjusted data (adjusted ∆805±2144 steps.day^-1^).

$ = within group differences analysed using a paired ttest

### Validation of the MID estimation

Three patients restarted a rehabilitation program, one patient was censored based on a cerebrovascular accident, two based on diagnosis of cancer (one sigmoid carcinoma, one pancreatic cancer), three patients were transplanted (two lung, one liver transplantation) and in two patients no reliable date of first hospitalization could be retrieved because of loss of contact after respectively four and six months.

**Fig 2:**
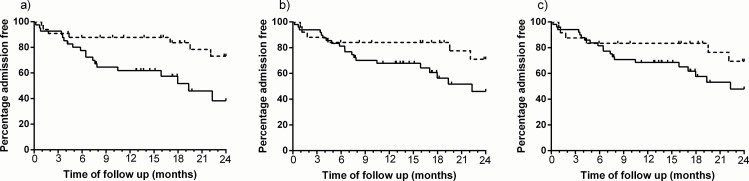
Time to first hospitalization. difference between patients exceeding the MID (dotted line) and patients not exceeding the MID (solid line), based on a) SEM cutoff, b) empirical rule effect size and c) cohen effect size and 0.5 SD.

During the mean (min-max) follow up period of 632 (341–720) days, 35% of participants had at least one hospital admission due to acute exacerbation. Time to first COPD admission in two years following the rehabilitation was shorter for patients not exceeding the MID according to the SEM (p = 0.01). Similar, though not statistically significant, trends were observed when using other cutoffs (Fig 2 and Table 5). Adjusting the analysis for lung function severity and baseline physical activity did not change the estimates (Table 5). Including all patients with at least two valid days of PA measurement (n = 86) did not change the results (S1 File).

**Table 5:** Time to first hospitalization between patients who exceed the MID and patients who don’t.

MID	Exceeding MID n (%)	HR (95%CI)	p-value	HR (95%CI)^a^	p-value ^a^
**SEM**	**31 (42)**	**3.08 (1.28–7.43)**	**0.01**	**3.07 (1.27–7.41)**	**0.01**
**Empirical rule effect size**	**25 (34)**	**2.24 (0.89–5.64)**	**0.09**	**2.24 (0.89–5.66)**	**0.09**
**Cohen’s effect size**	**24 (32)**	**2.03 (0.81–5.08)**	**0.13**	**1.99 (0.79–5.04)**	**0.15**
**0.5 times SD**	**24 (32)**	**2.03 (0.81–5.08)**	**0.13**	**1.99 (0.79–5.04)**	**0.15**

HR = hazard ratio, 95%CI = 95% confidence interval, SD = standard deviation

^a^ adjusted for baseline PA (STEPS_baseline_) and baseline disease severity (FEV1%pred_baseline_)

## Discussion

The present study provides an estimation (between 599 and 1131 steps.day^-1^) and clinical validation of the MID in PA for patients with COPD. Patients who exceeded the MID show a decreased risk for a hospital admission in the first two years after rehabilitation, providing validity to the proposed MID.

The present study included different distribution-based estimations, resulting in a range mainly varying based on the chosen SD in the calculation [25]. Because intervention studies focus on changes over time, calculations based on SD of the change seem more appropriate. The most established and used method is the SEM, which is expressed in the original metric of the instrument (steps) and is by definition sample independent [26]. Our results confirm the clinically meaningfulness of the 1-SEM criterion [26]. Other possible methods of MID estimation are including a global rating of change questionnaire (within patient) or interpatient comparison [27]. These techniques could not be included because of the retrospective design of this study. Pedometer-based interventions in COPD elicit a weighted increase of 562 steps per day [28]. More recently published coaching trials showed larger increases up to 3000 steps [29,30], showing the feasibility of the MID as described in the present study. Nevertheless, in e.g. pharmacological studies, aiming for an increase of 600 steps will still be challenging and require a decent sample size as PA measurements demonstrate large variability, even after proper standardization [18]. The availability of a Minimal Important Difference can assist the design and interpretation of future studies aiming to enhance PA. On one hand the MID can form the basis of the sample size calculation and on the other hand the MID will help the interpretation of findings. Ideally, new interventions should aim to increases exceeding the present MID estimate, which can then consequently, at the group level, be interpreted as clinically relevant. Alternatively, ‘number needed to treat’ can be calculated based on the available MID.

We selected time to first hospital admission as an important clinical indicator. The aim of this validation is only to evaluate whether a threshold, known to be statistically relevant, can be translated to clinical relevance and did not intend to identify independent predictors of admissions nor aimed to identify the best discriminant steps value for better clinical outcomes in general. Similarly, whether smaller increases would already have a positive effect on hospital admission or would lead to other clinical benefits (e.g. cardiovascular prevention) is not known but goes beyond the scope of this study. Nevertheless our data are in line with previous research concluding higher physical activity [31,32] and an increase of steps [33] being protective of severe acute exacerbations.

To the best of our knowledge the present study is the first identifying the MID in objectively measured PA in patients with COPD [8]. The MID in the London Chest Activity of Daily Living Scale has already been reported [34]. Multiple sclerosis is the only disease in which the MID has been calculated for steps. This MID lays in the same order of magnitude compared to the present study: 779 steps per day [35]. Although most recommendations of PA include other variables, we chose only daily step count as outcome measurement. Beside the fact that this variable is easy to understand and highly used in clinical practice, this choice was based on our previous data showing step counts to be a more sensitive outcome to detect changes [18]. In addition we used two activity monitors and step counts between both are sufficiently similar. This may not be the same for other outcomes that are relying on proprietary algorithms. Whether other outcomes of activity would correlate sufficiently with the proposed anchors and allow an estimation based on triangulation is not known and ground for further investigation.

Despite aiming to include multiple methods in the MID estimation and add a clinical validation, some limitations exist. This retrospective study included PA data measured with 2 (valid) activity monitors. Although previous research suggest the Sensewear Armband to be less accurate in measuring step counts [36], the correlation between day-by-day differences in step count obtained by both devices was high and both devices resulted in the same trends of differences in the validation of the MID (both in S1 File). A second limitation is the inclusion of two cohorts. Because these cohorts show comparable disease severity and PA levels, this would unlikely have changed the MID estimates. A third limitation is that we were not able to relate a minimal change to patient-important aspects due to the lack of anchor-based methodology. The present study provides further evidence to the concept that PA is a particular feature of patients’ health and is poorly captured by exercise tests and health status patient reported outcome tools. Because distribution-based approaches only provide an indication of the MID based on statistical criteria it has been highly recommended to add multiple anchor based-approaches in the estimation. Previous research concluded slightly better correlations between PA and our proposed anchors [21], however, also not reaching the requirements for anchor-based estimations. In addition, we validated the MID using a clinical important indicator, supporting the present results. Although the use of higher cutoffs did not reach statistically significant differences between patients exceeding the MID and patients who did not, we are confident that this analysis provides a decent validation because similar trends are observed at these cut-points. Research using larger samples would probably be able to confirm this. A possible bias of the presented study is the inclusion of patients referred for a multidisciplinary rehabilitation program, representing the rather inactive patients, as well as only including patients who successfully finished rehabilitation, which was crucial to obtain a valid estimate of changes in PA. Further research is needed in a cohort representing a broader spectrum of PA levels to confirm the present results.

Estimation of a MID is important in the interpretation of interventions as it goes beyond the interpretation of the concept of statistical differences. Besides giving an estimation of this meaningful increase, our data show the clinical importance of this change. As with all MIDs, individual patients may perceive a worthwhile benefit below or above this estimation, since these estimates are based on groups of patients. It should be noted that the MID possibly varies across patient groups [9] and that the obtained MID might be intervention-specific (if the variance of the effect would be different between interventions [37]). Our data would support that interventions yielding an increase of 600–1100 steps yield clinically important benefits to patients with COPD. Further research is needed to confirm the estimation in the PA in COPD, including other interventions, avoiding the bias of a single study estimation. As part of the IMI-JU PROactive project (www.proactivecopd.com) large intervention studies were conducted, investigating the effect of drugs (EudraCT 2013-002671-18), pulmonary rehabilitation (NCT02437994) and telecoaching interventions (NCT02158065) on physical activity. These studies, besides others, could help confirming the present results in different interventional paradigms.

## Conclusions

The Minimal Important Difference in daily step count after pulmonary rehabilitation, based on distribution-based calculations, lies between 600 and 1100 steps per day. An anchor-based approach could not be used because of the lack of well-related anchors. The clinical importance of this change is supported by a reduced risk for hospital admission in those patients with more than 600 steps improvement after pulmonary rehabilitation. Future research is needed to confirm this estimation based on data from one single center.

## Supporting Information

S1 File Online Supplement. Online data supplement including patient characteristics and additional data analyses including the relation between the 2 activity monitors and sensitivity analyses according to the validation of the MID estimation
(DOCX)

S1 Fig Relation between the 2 activity monitors. (TIF)

S2 Fig Time to first hospitalization including all patients with at least 2 days of measurement. (TIF)

S3 Fig Time to first hospitalization according to the activity monitor used. (TIF)
