# Supplementary material for: The Minimal Important Difference in Physical Activity in Patients with COPD
Source: PLoS One. 2016 Apr 28;11(4):e0154587. doi: 10.1371/journal.pone.0154587 (PMC4849755; doi:10.1371/journal.pone.0154587)
Supplement: S1 File — Online data supplement including patient characteristics and additional data analyses including the relation between the 2 activity monitors and sensitivity analyses according to the validation of the MID estimation (DOCX) [file pone.0154587.s001.docx]

# **The minimal important difference in physical activity in patients with COPD**

**Online supplement**

Patient characteristics
A total of 37 and 89 patients with COPD formed respectively the test-retest and rehabilitation sample of the present trial. Fifteen patients in the rehabilitation and 7 patients in the test-retest sample were excluded because of an insufficient number of valid PA days. There was no difference between included and excluded patients, in both cohorts (Table S1).

|  | Test-retest sample | | | Rehabilitation sample | | |
| --- | --- | --- | --- | --- | --- | --- |
|  | Included (n=30) | Excluded (n=7 ) | p-value^$^ | Included  (n=74) | Excluded  (n=15) | p-value^$^ |
| Age (years) | 67±7 | 68±8 | 0.65 | 66 ± 7 | 67 ± 7 | 0.48 |
| BMI (kg.m^-2^) | 27±6 | 25±5 | 0.45 | 26 ± 6 | 26 ± 6 | 0.64 |
| Gender (M/F)* | 23 (77%)/ (23%) | 6 (86%) / 1 (14%) | 0.60 | 55 (75%) / 18 (25%) | 12 (75%) / 4 (25%) | 0.98 |
| 6MWD (m) | 457 ± 113 | 425 ± 148 | 0.53 | 409 ± 120 | 451 ± 71 | 0.07 |
| FEV_1_ (%pred) | 48 ± 12 | 49 ± 13 | 0.91 | 48 ± 22 | 46 ± 16 | 0.76 |

**S1 Table**. **Baseline characteristics of patients included in and excluded from the present analysis**.
Data expressed as mean ± SD, *=data expressed as n (%); $= between group differences analyzed using an unpaired ttest or chi-square test(*)

Relation between the 2 activity monitors
PA was measured by the Sensewear Pro Armband (BodyMedia, Pittsburgh, PA, USA) in 41 patients in the rehabilitation and 11 patients in the test-retest sample and by the Actigraph GT3X (Actigraph LLC Pensacola, FL, USA) in 33 patient in rehabilitation and 19 patients in test-retest sample. Based on 9 patients in the test-retest cohort with valid data of both activity monitors (minimum 8 weekdays), a strong correlation (r=0.93, p<0.0001) could be observed between the day-by-day changes as measured by the Actigraph GTX3 and the Sensewear Armband (Fig S1). Day-by-day changes are calculated as the difference of the step count to the mean measurement (mean of all days measured by both devices), for each measurement day.

S1 Fig. Relation between the 2 activity monitors. This graph represent the difference of each day to the mean step count (mean of all data by both devices), as measured by The Actigraph GT3X and Senswear Armband. These day-by-day differences were strongly correlated (r=0.93, p<0.0001)

*Validation of the MID estimation*
***In patients with at least 2 days of measurement***The time to first hospitalization due to an acute exacerbation in 2 years following the pulmonary rehabilitation in 86 patients with at least 2 valid days of PA measurement is shown in Fig S2. In previous research, 2 days of PA measurement has been shown to result in a reliable measurement for cross sectional analyses. The results are comparable with those reported in the main analysis.

**S2 Fig.**  **Time to first hospitalization including all patients with at least 2 days of measurement.** Difference between patients exceeding the MID (dotted line) and patients not exceeding the MID (solid line) based on SEM cutoff (A), the empirical rule effect size (B) and the cohen effect size and 0.5 SD (C). *adjusted p-values.

***Analysis according to the physical activity monitor***
Time to first hospitalization between patients exceeding the MID and those not exceeding the MID in patients measured with Sensewear Pro armband (panel A-C) or Actigraph GT3X (panel D-F) is presented in Fig S3. 41 Patients were measured using the Sensewear Pro armband, 33 patients using the Actigraph GT3X. In both devices similar trends can be seen.

**S3 Fig**. **Time to first hospitalization according to the activity monitor used**. Difference between patients exceeding the MID (dotted line) and patients not exceeding the MID (solid line) based on the SEM cutoff (A), the empirical rule effect size (B) and the cohen effect size and 0.5 SD (C) in patients measured with Sensewear Pro Armband; on SEM cutoff (D), empirical rule effect size (E) and cohen effect size and 0.5 SD (F) in patients measured with Actigraph GT3X.
